# Prescription for a Healthy Journal

**DOI:** 10.1371/journal.pmed.0010022

**Published:** 2004-10-19

**Authors:** The PLoS Medicine Editors

## Abstract

Why does the world need a new medical journal? In this first editorial the *PLoS Medicine* Editors set out their vision for the journal

## Teaser

Take monthly, at no cost; reaches six billion

Today the possibilities for a medical journal are almost limitless. The first medical journals reflected the needs of a closed group of doctors. But medicine, its place in the world, and the dissemination of information have changed utterly. So in starting afresh, what should a new medical journal retain, and what should it ditch?

Most obviously, we should throw out the old way of disseminating information. In today's electronic age, it is no more difficult, and it is only minimally more costly, to provide access to one million people than it is to one person. So the revolutionary idea of anyone being able to read any article is possible. This idea—open access—which completely challenges the old subscription-based publishing model, is the driving force behind the launch of *PLoS Medicine*. You can download and distribute articles without restrictions (feel free to make a thousand copies, translate articles into other languages, put articles into books—just give the author proper credit).

We have also changed the way we involve the academic community in our journal. Our large global editorial board reflects the diversity of medicine today and is intimately involved in what we do. In particular, members of the editorial board are a crucial part of our peer review process. As academic editors they, along with a senior editor at the journal, take research papers through the peer review process in a way that we believe provides the most constructive and fair review. We are delighted that members of our editorial board have also shown their support for our journal by submitting papers to us, even before we launched.

What will we publish? The research article on malaria in this issue reflects our priority of publishing papers on diseases that take the greatest toll on health globally. But we will also publish papers reporting a substantial advance in any specialty, whether that advance is in public health, such as the paper on the global burden of disease; drug effects, such as the paper on the effect of HIV drugs on lipids; or the molecular understanding of disease, such as the paper dissecting out the immune responses in lung disease caused by smoking.

A good general medical journal should also be a place where the global medical community can discuss together what matters to them. The magazine section of *PLoS Medicine* will be devoted to comment, lively debate, and diverse opinions, in particular giving neglected voices and diseases a place in the limelight. In this issue's magazine section you will see articles from five continents that cover a huge range of topics, from basic sciences (such as the pathology of emphysema) to global public health (such as palliative care in developing countries). You will find diverse opinions—for example, on whether President Bush is helping or hindering Africa's progress towards tackling HIV, and on whether health professionals should routinely screen women for domestic violence (tell us what you think by taking our poll at www.plosmedicine.org). And you'll find case-based learning materials on meningitis linked to an online video and an online quiz.

The revolutionary idea of anyone being able to read any article is possible.

Interpretation of results is an essential part of a medical journal's job. Although we expect that many of our readers will be doctors, we hope readers will range from patients wanting to learn about the latest research on their illness, to teachers wanting to use an article in the classroom, to policymakers. Hence, we have several levels of comment on original research. Perspectives, written by an expert, are aimed at readers who are already familiar with the topic. Synopses, written by *PLoS Medicine*'s professional editors, should provide any health professional with a quick introduction to an article. Patient summaries provide a starting point for patients to assess the relevance to them of a research paper.

We have decided not to be part of the cycle of dependency that has formed between journals and the pharmaceutical industry, an industry that focuses overwhelmingly on the most profitable drugs, thus sidelining many of the world's health problems. Medical journals have allowed their interests to become aligned with those of the pharmaceutical industry by printing advertisements for drugs, publishing trials designed by drug companies' marketing departments, and making profits on reprints used as marketing tools. *PLoS Medicine* will not accept advertisements for pharmaceutical products or medical devices. Our open-access license allows free distribution of articles, so PLoS cannot benefit from exclusive reprint sales. And we consider as the lowest priority for publication papers that are simply aimed at increasing a drug's market share without obvious benefit to patients.

We will aim to have the highest levels of transparency in our published papers. We require authors to tell us of any possible competing interests; we in turn will tell readers about them.

But, information flow should not be just one-way. Our editorial doors (or at least our E-mail boxes) are always open. We want your feedback on the journal: send us an E-mail or submit an eLetter about any article in the journal, take part in our polls, contribute ideas for the magazine section and submit original research. *PLoS Medicine* is a journal for the global medical community; we invite you to join in.

